# Polyethylene Oxide (PEO) and Polyethylene Glycol (PEG) Polymer Sieving Matrix for RNA Capillary Electrophoresis

**DOI:** 10.1371/journal.pone.0123406

**Published:** 2015-05-01

**Authors:** Yoshinori Yamaguchi, Zhenqing Li, Xifang Zhu, Chenchen Liu, Dawei Zhang, Xiaoming Dou

**Affiliations:** 1 Institute of Photonics and Bio-medicine (IPBM), Graduate School of Science, East China University of Science and Technology (ECUST), 130 Meilong Road, Shanghai 200237, China; 2 Engineering Research Center of Optical Instrument and System, Ministry of Education, Shanghai Key Lab of Modern Optical System, University of Shanghai for Science and Technology, No. 516 Jungong Road, Shanghai 200093, China; 3 Department of Applied Physics, Graduate School of Engineering, Osaka University, Yamadaoka, Suita-city, Osaka, 565–0871, Japan; 4 College of Photoelectric Engineering, Changzhou Institute of Technology, No.299, Tongjiangnan Road, Changzhou, 213002, China; School of Applied Sciences—RMIT University, AUSTRALIA

## Abstract

The selection of sieving polymer for RNA fragments separation by capillary electrophoresis is imperative. We investigated the separation of RNA fragments ranged from 100 to 10,000 nt in polyethylene glycol (PEG) and polyethylene oxide (PEO) solutions with different molecular weight and different concentration. We found that the separation performance of the small RNA fragments (<1000 nt) was improved with the increase of polymer concentration, whereas the separation performance for the large ones (>4000 nt) deteriorated in PEG/PEO solutions when the concentration was above 1.0%/0.6%, respectively. By double logarithmic plot of mobility and RNA fragment size, we revealed three migration regimes for RNA in PEG (300-500k) and PEO (4,000k). Moreover, we calculated the smallest resolvable nucleotide length (*N*_min_) from the resolution length analysis.

## Introduction

Capillary electrophoresis(CE) is a powerful technology for nucleic acids separation.[1] For the separation of DNA/RNA fragments, especially the longer ones, CE filled with polymer solutions is the only available method for high-throughput analysis. Although microchip has been utilized for DNA sequencing, CE is still a standard and fundamental analytical instrument for DNA sequencing, besides CE is simple and low cost.[2,3] Especially when CE is combined with fluorescence detection method, a widely employed technology in bioanalytical science[4,5,6,7], it will be of great sensitivity. Capillary polymer electrophoresis, in which the polymer solutions were filled in capillary tube, still needs the fundamental investigations, including the direction of high voltage, polymer solutions, buffer solutions,[8,9] and migration process[10,11,12] for the application of hyphenated biological applications[13,14,15], and for RNA fragment separation.

The ratio of charge to nucleic base is uniform for both DNA and RNA. Thus in CE, the separation of nucleic acids requires the sieving matrix to differentiate the migration time. The polymer solution, which is usually the linear polymer solution, is used as the sieving matrix for DNA/RNA separation. When the nucleic acid migrates through the polymer solution, it entangles with the polymer chains. As different size of nucleic acid molecule has the different interaction of entanglement with polymer chains, the migration time is differentiated according to the length of nucleic acid size. Thus the migration process, which is dominated by the entanglement process between the polymer chains and DNA/RNA fragments, is different according to the sample size.[12,16,17]

DNA migration process in electrophoresis has been theoretically and experimentally studied very well in the past few years. [18,19,20,21,22,23,24] In the theory of DNA migration in polymer solutions, there are several regimes for DNA migration process.[20,21,22,25] Each regime is resulted from the migration morphology of the DNA during electrophoresis. The separation regime is also strongly depended on the physical properties of the sieving polymer, such as concentration, polymer length, and polymer chemical compositions.

The migration processes in polymer matrixes are also strongly decided by the properties of the analyte. RNA and DNA are physically deferent molecules and also have many similar properties. Because the sieving process of the DNA/RNA in polymer solution dominates the migration process of them during CE, the stiffness of the DNA or RNA are important physical properties for understanding the DNA/RNA separation in CE. Since the persistence length of DNA is about 20 nm while RNA is about 3 nm,[26,27] the sieving process of the RNA is different from the sieving process of DNA in CE. The approach to further increase the high throughput of RNA analysis is the identification of the sieving matrixes. For polymer solutions, several physical characteristics, including the high-separation performance, excellent chemical stability to buffer solution, low background for fluorescence detection, ease of preparing the polymer solutions, are considered for a high performance and excellent reproducibility of CE.

Polyethylene glycol (PEG) and polyethylene oxide (PEO) were well characterized by the studies of physical[28,29], micelle formation[30], rheological phenomena[31], solution dynamics [32] and morphology[33]. PEO was always employed for DNA sequencing by filling into capillary tubes[34]. PEG/PEO were also employed for the DNA sequencing because the PEO solution possessed homogeneous structure. They were easy to prepare, and were able to provide high resolution power for DNA separation in both direct current (DC) capillary electrophoresis and alternative current (AC) capillary electrophoresis. [35]

In this paper, we demonstrated the separation of RNA (100–10,000 nt) CE to investigate the separation performance of RNA. The sieving polymers were PEG and PEO with different molecular weight: 300–500 k and 4,000 k. We found that the resolution length of RNA was ranging from 6.0 nt to 3522.8 nt and the best length resolution was 6.0 nt in the solution of PEO at 0.8% (w/w).

## Material and Methods

### Chemicals

PEG of average molecular weight (Mw) of 4 k in powder was purchased from Sigma-Aldrich, Co. (Atlanta, USA). PEG(300–500 k) and PEO (4,000 k) powders were purchased from Wako Pure Chemical Industries Ltd (Osaka, Japan). The polymer solutions were prepared by adding the polymer powder, TBE powder (TAKARA, Shanghai, China) and urea powder (Sinopharm Chemical Reagnent Co., Ltd, Shanghai, China) into ultrapure water, and then the solutions were stirred for more than 12 hours.[35] Then the homogenous polymer solutions were degased using a vacuum pump. Finally, the polymer solutions were diluted to different concentration and applied to the CE system. The RNA marker (TAKARA BIO INC., Dailian, China) were diluted to a solution of 350 ng/μl concentration, which also contained 4.0 M urea and 0.5× TBE. Prior to CE, RNA sample was denatured by 4.0 M at 65°C for 5.0 min, and then was fast cooled on ice for 3.0 min. SYBR Green II were bought from Invitrogen (Carlsbad, CA, USA).

### Capillary Electrophoresis

The CE system had been described in previous works.[36] The total length and effective length of the 75μm ID fused-silica capillary (Polymicro, Yamato, USA) were 9 cm and 6 cm, respectively. It was coated by S. Hjertén method to suppress the electroosmotic flow (EOF).[37] A high-voltage power supply was employed to provide high voltage between the two ends of the capillary. The exciting light from a mercury lamp was filtered by an optical filter (U-MWIB3, Olympus, Japan) to a wavelength of 460–495 nm, which was the excitation wavelength of the conjugate of SYBR Green II and the nucleic acid. The excited fluorescence emission was collected by a 100× objective (MPlanFL N, Olympus). A photomultiplier tube (H8249-101, Hamamatsu Photonics, Japan) was used to detect the collected light. LabVIEW software (National Instrument, Austin, TX, USA) was applied for high-voltage power supply control and data collection. After each run, the capillary was flushed with ultrapure water by a vacuum pump. The whole CE detection proceeded in a dark box under room temperature.

### Resolution and resolution length (RSL) of the peaks

The resolution (Rs)[38] was determined by the following equation: Rs = 2 Δx / (w_1_ + w_2_). Here, Δx is the difference of the migration time of the concessive two peaks in electropherogram and w is the time of the full width of maximum of the peak (w_1_) and following peak (w_2_). The Gaussian curve fitting was performed by Origin Pro 8 SR0. The author’s observation was applied to find the width where the RNA peaks in electrophoretogram with considering the tailing, especially the peaks corresponding to the RNA longer than 1,000 nt, consistently appearing in the all experiment.

The resolution length (RSL) was determined by the following equation: RSL = Δn / Rs, where Δn is the RNA fragments length difference of adjacent peaks in electrophoretogram and Rs is the resolution. RSL is for the determination of the minimum RNA nucleotide length, which are the base pairs that can be separated with baseline separation in which the resolution is equal to 1.

## Results and Discussion

The fundamental structures of PEG and PEO are the same, because both of them are the polymers of ethylene oxide. The polymer of ethylene oxide with the molecular weight above 20,000g/mol is PEO and the one with molecular weight below 20,000 g/mol is PEG. We prepared PEO (4,000 k) and PEG (300–500 k), PEG(4 k) for our experiment, and observed the cognizable peaks, which correspond to the RNA ladders migration in the above polymer solutions.

The purpose of the investigation of the RNA migration in PEG/PEO solution is to find the regimes of the RNA sieving process in these polymer solutions and the quality of the separation, which is quantified by the minimum resolvable difference of the nucleotide length. PEG(4 k) was tested as the sieving matrix for CE first, but no recognizable peak appeared in the electrophoretogram when PEG polymer concentrations were 5%,10%, 15%, and 20%.

**Fig 1:**
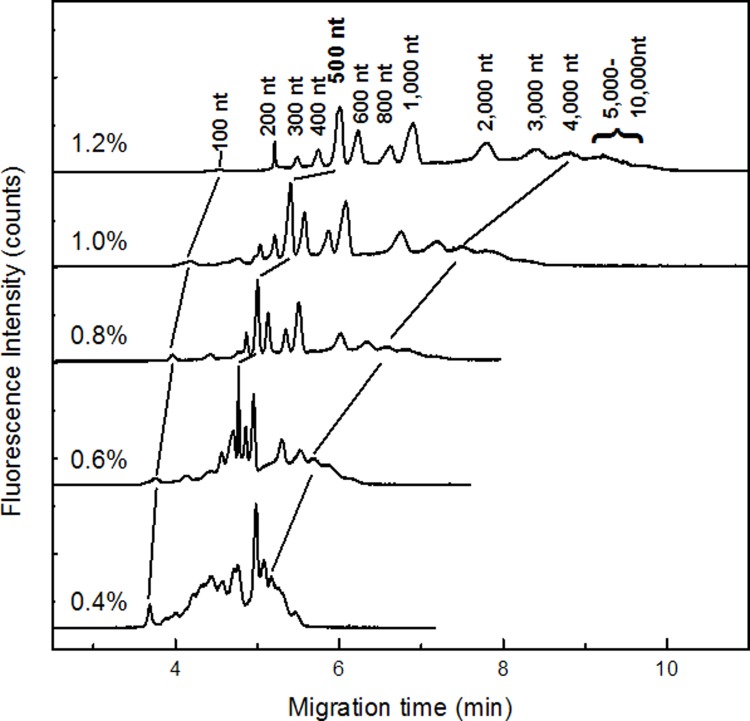
Capillary electrophoresis of 14 RNA fragments sized from 100 to 10,000 nt in PEG solution (300k-500k) with concentration varied from 0.4% to 1.2%. Capillary electrophoresis was performed at 100V/cm. The total length and the effective length of the capillary were 9.0 cm and 6.0 cm, respectively. Sample Loadings: 100 V/cm (2.0 sec).

We then performed the separation of RNA in PEG(300–500 k) with various concentrations, and the polymer solution contained 4.0 M urea. The RNA sample was denatured by 4.0 M urea prior to injection. Data in Fig 1 demonstrated that RNA ranging in size from 100 nt to 3,000 nt was baseline resolved when the polymer concentrations were 1.0% and 1.2%, but the longer RNA (> 5000 nt) can hardly be observed, which was possibly because the volume of them was too little. For diluted PEG solutions (0.6% and 0.8%), RNA sample shorter than 4,000 nt could be separated within 7.0 min, although the resolution for the adjacent RNA fragments was not better than they were in the concentrated solutions. For the more diluted PEG solutions (<4.0%), the peaks in the electropherogram were overlapped, and thus they cannot be resolved. We also attempted the RNA separation in the more concentrated PEG solutions (2.4% and 3.6%), but found that no recognized peaks appeared in the electropherogram. Moreover, compared with the RNA migration in hydroxyethyl cellulose (HEC) solution[10,39,40], it seems that RNA moves much faster in PEO solution than it is in HEC solution.

**Fig 2:**
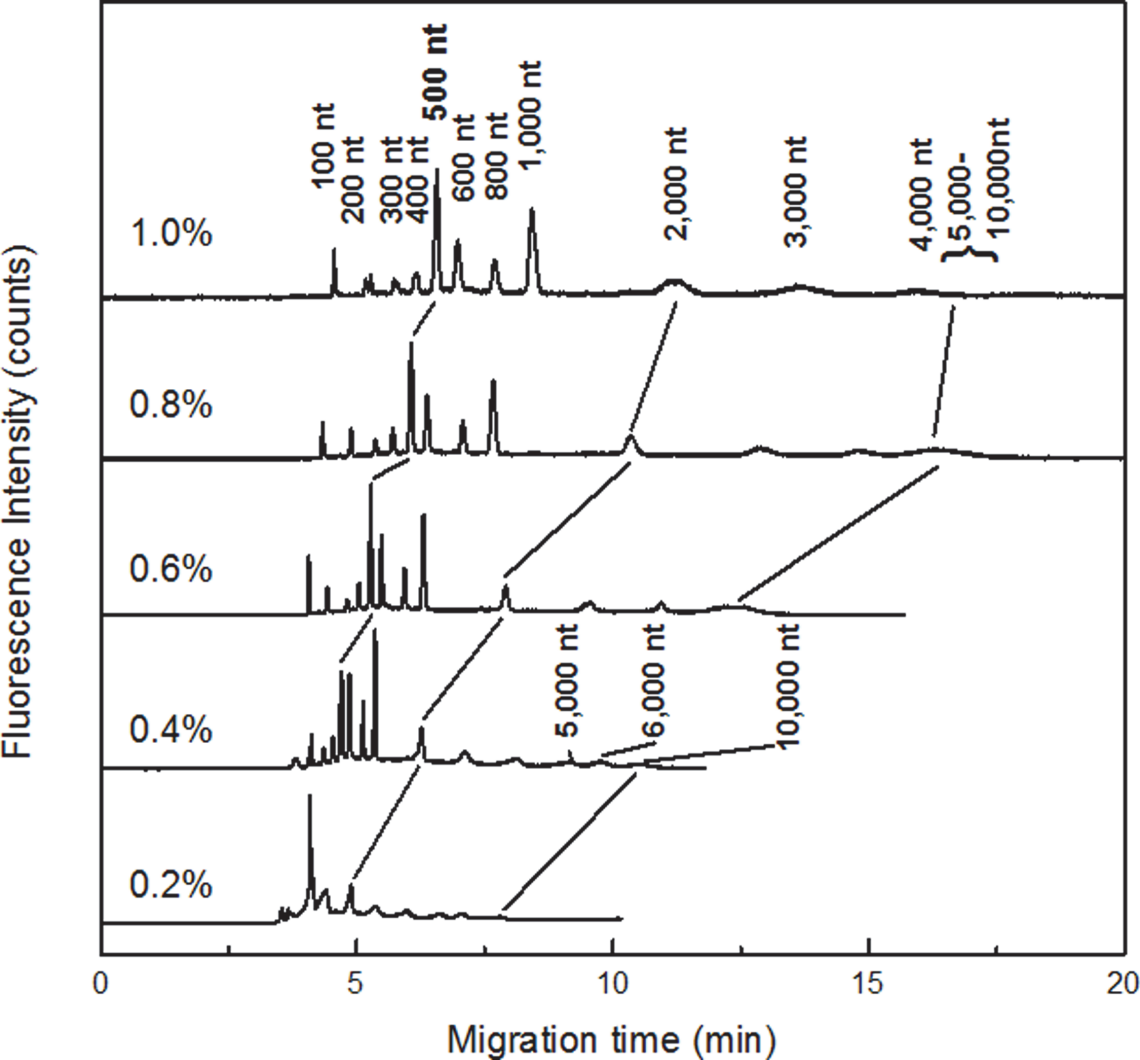
Capillary electrophoresis of 14 RNA fragments in PEO solution (4,000k) with concentration varied from 0.2% to 1.0%. Sample and capillary electrophorrtic conditions were the same as those in Fig 1.

We repeated the same experiment for RNA separation in PEO(4,000 k) solutions by CE, and the results were demonstrated in Fig 2. It showed that all 14 RNA fragments were resolved in 0.4% PEG solution, this was most possibly because the length of the polymer in PEO (4,000 k) was longer than that of PEG (300–500 k). When the concentration of PEO increased from 0.4% to 1.0%, only RNA fragments from 100 nt to 4.000 nt were baseline resolved. Comparing the separation performance of RNA in PEG(300–500 k) with that in PEO(4,000 k), the long RNA fragments (4,000, 5,000, 6,000 and 10,000 nt) were separated only in 0.4% and 0.2% PEO solutions, which has a higher molecular weight, indicating that PEO solution yielded better solution for longer RNA fragments. RNA fragments from 100 nt to 1,000 nt were also well separated in PEO solutions concentrated from 0.4% to 1.0%. This was because the radii of gyration of the RNA molecules had the same order of magnitude with the pore size of the polymer matrix. [41,42] Thus we can concluded that the higher concentration of PEO solution served the higher performance for the shorter RNA, and the lower concentration of PEO solution favored longer RNA. We also attempted the RNA separation in 1.5% and 2.0% PEO polymer solution, but we observed only slight peaks of 100–1,000 nt RNA fragments in 1.5% PEO solution and no separated peaks in 2.0% PEO solution (not showed in the Figures). This might because the pore size of the network in these concentrated polymer solutions was too small to allow the RNA fragments get through.

**Fig 3:**
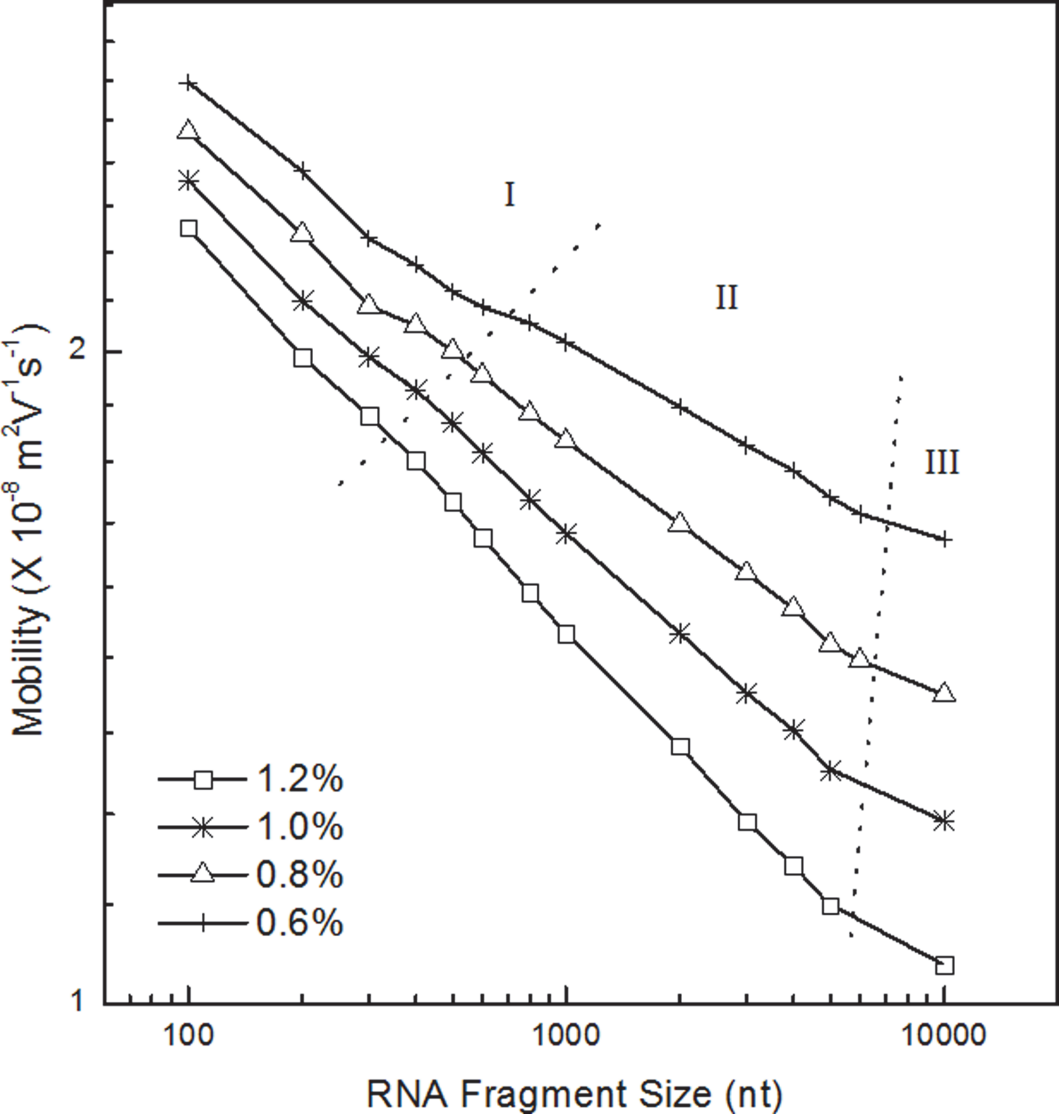
The double logarithmic plot of the mobility versus RNA fragment size in PEG with average molecular weight of 300k – 500k. The plot was prepared based on the electrophoresis in Fig 1.

**Fig 4:**
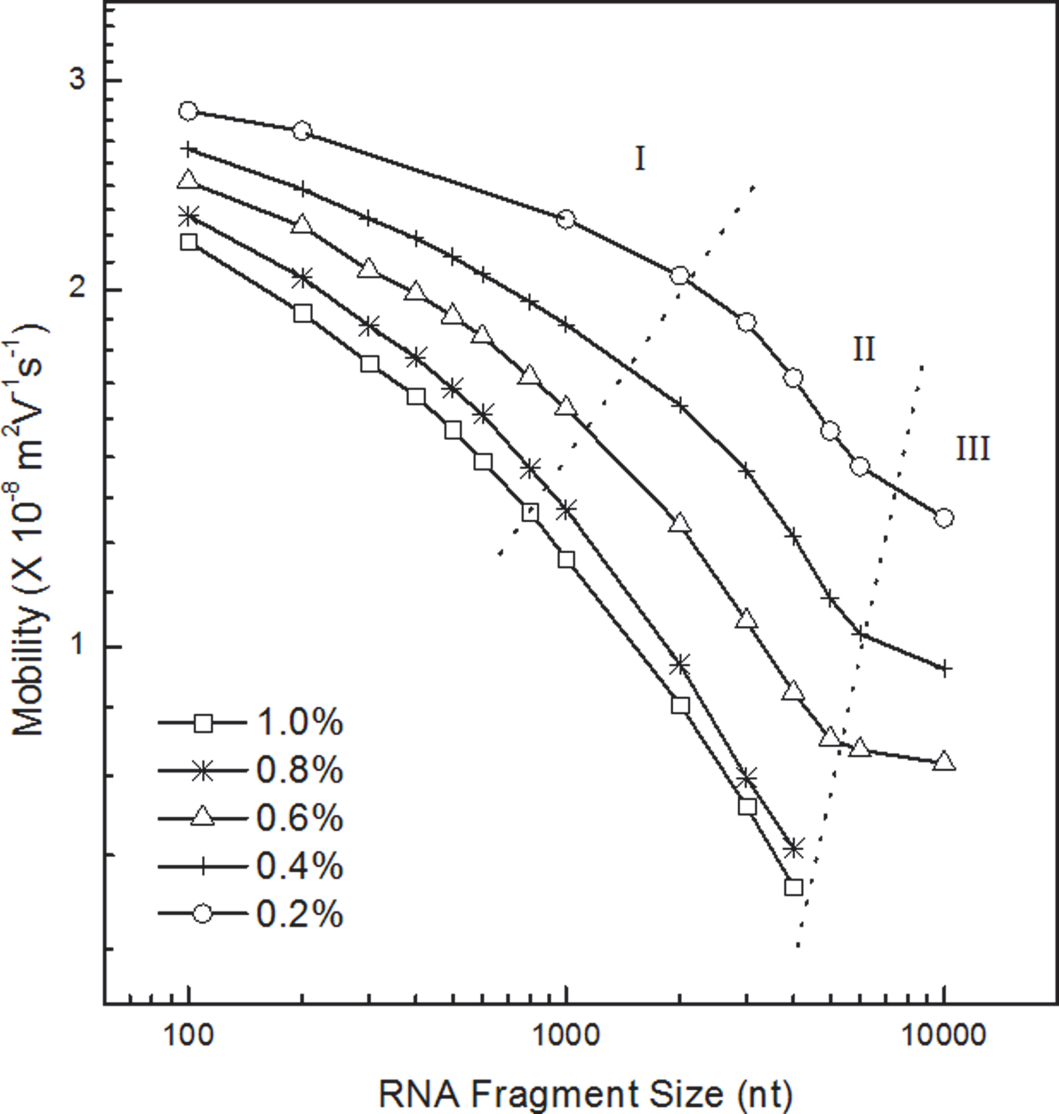
The double logarithmic plot of the mobility versus RNA fragment size in PEO with average molecular weight of 4,000k. The plot was prepared based on the electrophoresis in Fig 2.

The migration patterns of RNA in PEG (300–500 k) solution was evaluated by double logarithmic plot of the RNA length and mobility (Fig 3). Three regimes were observed. They were Ogston (section I), reptation regime (section II) and reptation with orientation (section III), and were consistent with DNA migration in polymer solution. [43] The threshold of the regime was around 300 nt to 700 nt which was approximately 90 nm. Because the radius of gyration of PEO polymer was estimated at about 40 nm, the point of the changing the model was justified.[41] And in the experiment of 4,000 k PEO solution, the same plot (Fig 4) was prepared to find the regimes. Three regimes were found from the plot and those were corresponding to Ogstion, reptation, reptation with orientation.

**Fig 5:**
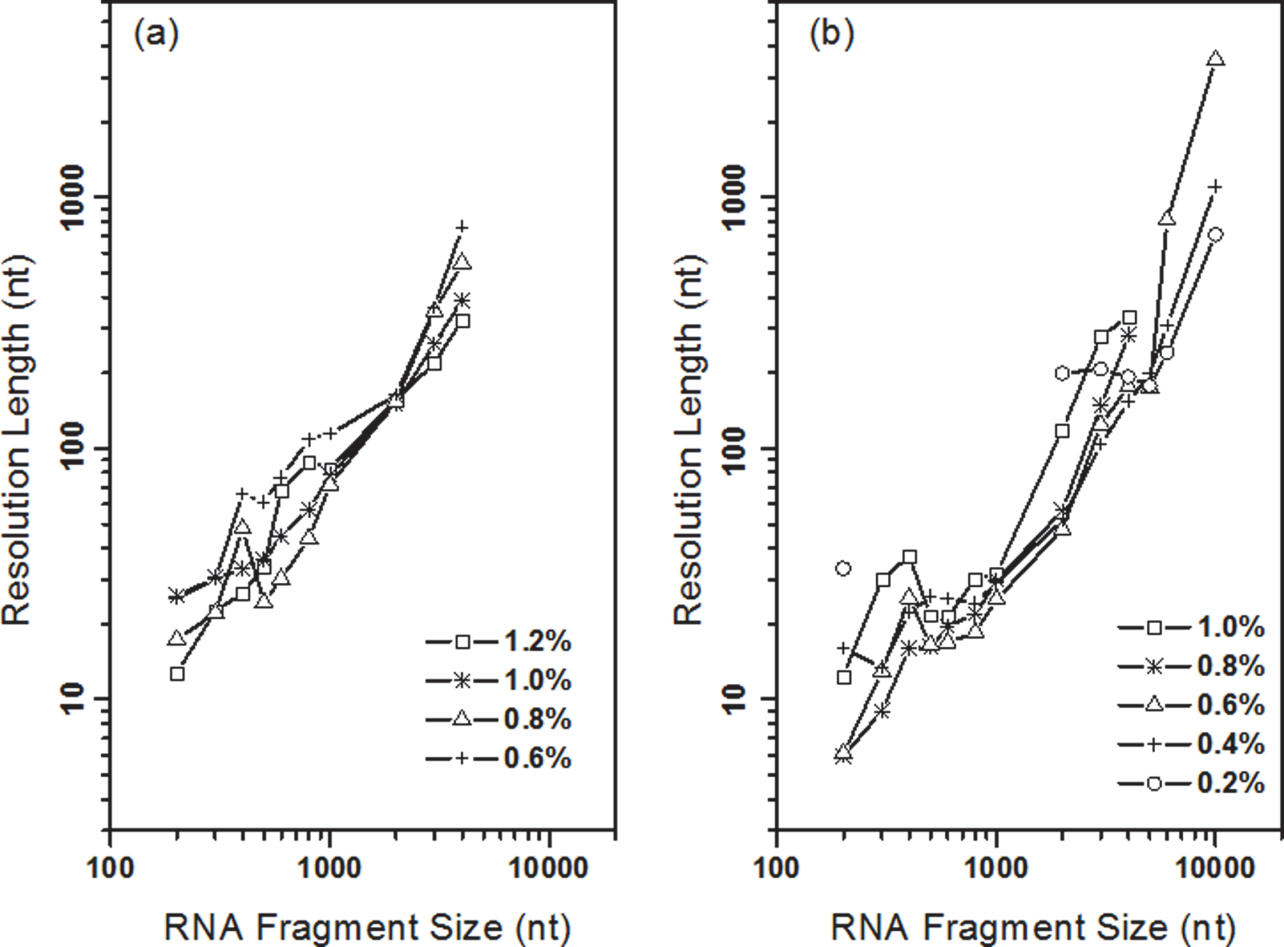
Resolution length of capillary polymer electrophoresis. (a) 300k-500k PEG polymer solution, (b) 4,000k PEO polymer solution. The plot of resolution length was prepared based on the electrophoresis in Figs 1 and 2.

The separation performance of RNA was also evaluated by the resolution length (Fig 5). Resolution length tells the minimum, which is smallest RNA nucleotides length resolvable with baseline separation. Theoretically, two adjacent RNA fragments with 1 nt difference in size could be resolved if the resolution length is equal to 1. The resolution length of RNA in PEG(300–500 k) was tabulated in Table 1. It showed that the resolution length varied from 12.7 nt to 761.4 nt, and the best, minimum resoluble nt was 12.7 nt for RNA between 100 nt and 200 nt. (Fig 5A) It was greatly improved than the resolution length (30.2 nt) observed in HEC solution by pulsed filed CE[39]. Because the minimum number (*N*_min_) was the nucleotide when the resolution was 1, the resolution 0.02 was required for 1 nucleotide deference separation. The resolution length for RNA in PEO(4000 k) was tabulated in Table 2, and it demonstrated that the resolution length (Table 2) varied from 6.0 nt to 3522.8 nt, where the best, minimum resoluble nucleotide length was 6.0 nt between 100 nt to 200 nt. (Fig 5B) For the separation of RNA fragments below 1,000 nt the PEO with 4,000 k showed better performance. Furthermore, we can find from Table 1 and Table 2 that the threshold in the migration regime moves to the short RNA fragments when the concentration of the polymer increases.

**Table 1:** The approximated threshold of the sieving process in each concentration of 300k - 500k PEG polymer and the smallest resolvable size of RNA estimated from the resolution length.

PEG polymer concentration	First point (nt)	Second point (nt)	BRL in first regime (nt)	BRL in second regime (nt)
1.2% (300-500k)	320–360	5,610–6,210	12.7	26.5
1.0% (300-500k)	390–450	5,980–6,620	25.8	33.1
0.8% (300-500k)	500–566	6,320–7,000	17.3	24.4
0.6% (300-500k)	670–750	6,850–7,550	25.3	76.6

Best resolution length was abbreviated as BRL.

**Table 2:** The approximated threshold of the sieving process in each concentration of 4,000k PEO polymer and the smallest resolvable size of RNA estimated from the resolution length.

PEO polymer concentration	First point (nt)	Second point (nt)	BRL in first regime (nt)	BRL in second regime (nt)
1% (4,000k)	740–820	More than 5,000	12.4	30.1
0.8% (4,000k)	840–930	More than 5,000	6.0	29.6
0.6% (4,000k)	1,070–1,180	5,040–5,580	6.1	47.7
0.4% (4,000k)	1,380–1,530	5,830–6,450	13.4	52.2
0.2% (4,000k)	2,000–2,210	6,970–7,710	33.2	177.1

## Conclusions

We performed a systematic research on the separation of RNA in PEG/PEO polymer solution with different molecular weight and concentration, and analyzed the separation performance by resolution length. We found three regimes of the RNA migration by the logarithmic plot of mobility *versus* RNA length in both PEG and PEO solutions. The polymer concentration affected the migration time, resolution between RNA fragments. In addition, the minimum resolution length for RNA in the optimal electrophoretic conditions, including the sieving polymer molecular weight, concentration, electric field strength and capillary length, was about 6.0 nt. Those perceptions for PEG/PEO sieving polymers motivated CE as an effective tool for RNA separations.
