# Correction: Polyethylene Oxide (PEO) and Polyethylene Glycol (PEG) Polymer Sieving Matrix for RNA Capillary Electrophoresis

**DOI:** 10.1371/journal.pone.0131265

**Published:** 2015-06-18

**Authors:** Yoshinori Yamaguchi, Zhenqing Li, Xifang Zhu, Chenchen Liu, Dawei Zhang, Xiaoming Dou

There are errors in the author affiliations. The affiliations should appear as shown here: Yoshinori Yamaguchi^1,2,3^, Zhenqing Li^2^, Xifang Zhu^4^, Chenchen Liu^2^, Dawei Zhang^2^, Xiaoming Dou^1^

^1^ Institute of Photonics and Bio-medicine (IPBM), Graduate School of Science, East China University of Science and Technology (ECUST), 130 Meilong Road, Shanghai 200237, China

^2^ Engineering Research Center of Optical Instrument and System, Ministry of Education, Shanghai Key Lab of Modern Optical System, University of Shanghai for Science and Technology, No. 516 Jungong Road, Shanghai 200093, China

^3^ Department of Applied Physics, Graduate School of Engineering, Osaka University, Yamadaoka, Suita-city, Osaka, 565–0871, Japan

^4^ College of Photoelectric Engineering, Changzhou Institute of Technology, No.299, Tongjiangnan Road, Changzhou, 213002, China
